# Metagenomic Studies of Viruses in Weeds and Wild Plants: A Powerful Approach to Characterise Variable Virus Communities

**DOI:** 10.3390/v13101939

**Published:** 2021-09-27

**Authors:** Beata Hasiów-Jaroszewska, Dieke Boezen, Mark P. Zwart

**Affiliations:** 1 Department of Virology and Bacteriology, Institute of Plant Protection-National Research Institute, Węgorka 20, 60-318 Poznań, Poland; 2 Department of Microbial Ecology, Netherlands Institute of Ecology (NIOO-KNAW), Droevendaalsesteeg 10, 6708 PB Wageningen, The Netherlands; D.Boezen@nioo.knaw.nl (D.B.); M.Zwart@nioo.knaw.nl (M.P.Z.)

**Keywords:** HTS, viromes, weeds, wild plants, plant virus

## Abstract

High throughput sequencing (HTS) has revolutionised virus detection and discovery, allowing for the untargeted characterisation of whole viromes. Viral metagenomics studies have demonstrated the ubiquity of virus infection – often in the absence of disease symptoms – and tend to discover many novel viruses, highlighting the small fraction of virus biodiversity described to date. The majority of the studies using high-throughput sequencing to characterise plant viromes have focused on economically important crops, and only a small number of studies have considered weeds and wild plants. Characterising the viromes of wild plants is highly relevant, as these plants can affect disease dynamics in crops, often by acting as viral reservoirs. Moreover, the viruses in unmanaged systems may also have important effects on wild plant populations and communities. Here, we review metagenomic studies on weeds and wild plants to show the benefits and limitations of this approach and identify knowledge gaps. We consider key genomics developments that are likely to benefit the field in the near future. Although only a small number of HTS studies have been performed on weeds and wild plants, these studies have already discovered many novel viruses, demonstrated unexpected trends in virus distributions, and highlighted the potential of metagenomics as an approach.

## 1. Introduction

Metagenomics is the analysis of environmental samples using high-throughput sequencing (HTS), with the intent of characterising microbial communities. HTS has the advantage of the identification and genomic characterisation of viruses in a target-independent manner, and it has proved to be a valuable tool for virus detection, discovery, or diversity studies. Classic virus detection methods, such as the enzyme-linked immunosorbent assay (ELISA) or polymerase chain reaction (PCR), usually target one or a few viral species and require prior knowledge of the virus to be detected, while HTS methods have been developed to look for virus-like sequences without the bias of only detecting known viruses. HTS technologies allow a generic approach to virus identification and can deliver species, or even strain-specific results [1]. There are several applications for metagenomics approaches in plant virus diagnostics: (i) identifying the causes of viral diseases in economically important crops; (ii) screening for specific viruses when their presence is suspected, (iii) detecting asymptomatic or cryptic viruses, and (iv) discovering novel viruses whose presence has not been reported before. To date, the majority of metagenomics studies have focused on economically important plant species, whereas the occurrence and population structure of viruses in weeds and wild plants have remained largely unstudied. Wild plants are important players in many biotopes—including environments with significant anthropogenic impact such as agroecosystems—and they have a great impact on virus epidemiology. Plant viruses can spill over in both directions between wild plants and crops with potential adverse effects in both managed and natural ecosystems. Knowledge about the occurrence and diversity of viruses in wild plants can offer insight into factors that promote long-term coexistence between hosts and viruses in nature, as well as the evaluation of the disease emergence risk [2].

In this review, we focus on the role of weeds and wild plants in virus emergence, and discuss recent metagenomic studies of weeds which show the potential of this approach for untargeted virus detection in wild hosts. We review recent discoveries of plant viruses inhabiting various ecological niches and discuss “pros and cons” of using metagenomics for this purpose. Moreover, we show the potential of using HTS to explore the diversity and richness of the viruses associated with plant populations. Finally, we discuss the future directions of plant virus metagenomics in weeds and wild plants.

## 2. Role of Weeds and Wild Plants in Virus Emergence

In agroecosystems, crop plants often grow side by side with weeds and wild plants. These wild plants constitute potential reservoirs for viruses that may spread into cultivated plants, thereby leading to epidemics or to the emergence of novel viruses [3,4,5,6]. Many viruses are known to have wide host ranges encompassing both wild plants and crops [3,7,8]. Viruses can emerge in cultivated plants from wild hosts or, conversely, virus infections in crops may spill over and affect wild plant populations. Spread from reservoirs into a new environment, establishing productive infections, and effective between-host transmission are necessary steps for virus emergence to occur [4]. Most plant viruses are transmitted by insect vectors, which may increase the possibilities for virus transmission across landscapes and the distances over which viruses can be transmitted [9,10,11]. Knowledge of virus diversity, prevalence, and dynamics in wild plant populations is, therefore, relevant to a better understanding of virus epidemiology and emergence in crops. Conversely, viruses in natural systems and viruses spilling over from agroecosystems could have a major impact on natural ecosystems [12,13]. Viruses in wild plants have been reported to be diverse and often do not induce evident symptoms in their host [13,14]. Therefore, these viruses may have been overlooked when sampling strategies focused on plants displaying overt virus symptoms [5,15]. For example, only 2.3% plant samples collected in the Oklahoma Tallgrass Prairie Preserve displayed symptoms suggesting viral infection, but using metagenomics, viruses were discovered in 25% of samples [16]. Similarly, large scale metagenomic studies conducted in France, America and South Africa revealed the presence of virus-related sequences in 25–58% of samples where the majority of plant specimens did not have outward signs of viral infection [17]. Across ecosystems, virus prevalence is observed to be high in wild plants, regardless of the presence or absence of symptoms [2,8].

### 2.1. Mixed Infection in Wild Plants

In wild plants, mixed infection—that is, the coinfection of a single plant by two or more different viruses—is common. These infections often include both acute and persistent viruses [5]. Mixed infection leads to a variety of intra-host virus–virus interactions, many of which may result in the generation of novel genetic features and subsequently change the genetic structure of the viral population. Recombination can lead to the generation of new variants, and segmented or multipartite viruses can form new variants through reassortment [18]. Like the distribution of fitness effects for other mutation classes, the majority of recombinants will be deleterious [19] but occasionally a new variant may be fitter or more virulent than the parental virus [20]. The interactions between viruses infecting a single host plant can be neutral, synergistic, or antagonistic with respect to virus accumulation (i.e., titer), host response (disease symptoms), or transmission efficiencies by vectors [20,21]. From crop systems, we know that virus–virus interaction can impact the prevalence and characteristics of infection. One example of this is the interactions between Parsnip yellow fleck virus (PYFV), Anthriscus yellows virus (AYV) and aphid vectors including *Cavariella aegopodii.* PYFV can be transmitted to carrot (*Daucus carota*) by aphids only when they have first acquired AYV. Carrot is not susceptible to infection by AYV, but introduction of the virus complex into carrot crops leads to scattered infections because individual aphids retain the transmission-dependent combination for only a few days [22], hereby increasing the effective host range of PYFV. The exchange of different virus components, including capsid proteins and helper components [9], could enhance transmission of viruses by mixed infection. Sharing of virus components in mixed infection may have far reaching and unexpected consequences. For example, a recent report suggests that Tobacco mosaic virus (TMV) infection enables a productive infection of tobacco plants by Cryphonectria hypovirus 1 (CHV1)—a fungal virus—because TMV’s movement protein allows CHV1 to move from cell to cell [23]. While metagenomics studies have shown mixed infection is common in wild plants, most of the work on the phenotype resulting from the interaction between viruses has been carried out in crop systems rather than wild plants.

### 2.2. Novel Viruses and Viral Diversity in Wild Plant Populations

Metagenomics has revolutionised plant virology by enabling the untargeted, simultaneous detection of multiple viruses regardless of their genomic nature [24]. A number of HTS-based approaches for identifying viral genomes have been developed using a wide range of nucleic acid targets. Workflows that characterise total nucleic acid, double-stranded RNA (dsRNA), virion-associated nucleic acids (VANA) purified from virus-like particles, ribosomal-RNA-depleted RNA and messenger RNA have been developed for this purpose [25]. In addition, the sequencing of small RNAs from the host has also been used for virus detection [17]. The small RNA approach works well for detecting viruses in a range of hosts, as one important immune response against viruses is RNA silencing, where virus-specific double-stranded RNA that is generated in most virus infections triggers a plethora of host responses that result in the sequence-specific degradation of RNA [26]. Using this method made it possible to discover novel viruses from both insects and plants [27,28]. An important limitation of small RNA sequencing is that it fails to detect viruses that either do not trigger silencing responses or that induce effective silencing suppression (e.g., persistent viruses) [17], and sequence assembly can result in chimeric genomes. Although more approaches and tools for metagenomics are becoming available, the number of studies addressing viruses occurring in weeds and wild hosts is still limited.

The majority of viruses found in natural systems have not been previously described [8,13]. Novel viruses are being discovered readily in wild hosts in diverse natural ecosystems [29], giving insight into virus biodiversity and the distribution of plant viruses across the globe [2,8,13]. Though the number of metagenomic studies in weeds and wild plants is increasing, the proportion of novel viruses uncovered in these plants remains high, indicating that there are many viruses yet to be identified and described in wild plant ecosystems. Besides the many new viruses detected, virus metagenomic studies have also revealed considerable variation in viral prevalence and community composition [2,6,8]. It has been suggested that particular virus families may be associated with crops, whereas other virus families would be associated with native vegetation. Thus, virus families characterised from crops may be quite different from those in wild plants [13]. These differences raise questions about the evolutionary forces and drivers acting on these different virus populations, their effects on viral adaptation to different hosts, and ultimately their effect on the assembly of viral communities. Metagenomic studies are now being used to answer these questions and analyse (1) the spatial distribution and genetic diversity of viruses in different landscapes; (2) the contribution of changes in plant populations and the effects of ecosystem simplification on viral pathogenicity or emergence, and (3) the dynamics of spillover from reservoirs to other hosts, an important component of the intensity and directionality of fluxes of viruses between crops and wild plants [6,8,30].

An overview of studies using metagenomic approaches for virus detection and diversity in weeds and wild plants is presented in Table 1.

Since 2010, studies using HTS to identify plant viruses have been conducted in 20 African countries, reviewed by Ibaba et al. [35]. At least 29 host plants, including various economically important crops, ornamentals, and medicinal plants representing 18 different families have been used in HTS for virus detection and diagnostics [32,35]. The studies resulted in the detection of previously known and novel viruses from almost any host, confirming the wide distribution of plant viruses in different ecosystems and suggesting the importance of knowledge on the diversity, prevalence, and spatial distribution of viruses. For example, four novel circular replication-associated protein (Rep)-encoding single-stranded (CRESS) DNA viruses were detected in wild and medicinal plant samples from *Poaceae* and *Apiaceae* species collected in Darling in the Western Cape region of South Africa [32].

Recently, HTS has also been applied by Minicka et al. [34] for the detection and identification of different viral species occurring in single and mixed infections in plants in Poland. The authors analysed 50 samples collected from different plant species during 2018–2019, including cultivated and ornamental plants, weeds, and trees. An interesting finding was made for weeds sampled from the areas adjacent to cultivated fields. HTS allowed for the identification of Clover yellow mosaic virus (ClYMV) (*Potexvirus* genus, Alphaflexiviridae family) on *Verbena officinalis* plants, and Melandrium yellow fleck virus (MYFV) (*Bromovirus* genus, Bromoviridae family) on *Silene latifolia* plants, whose presence has not been reported in Poland before. ClYMV infects broad bean, pea, alfalfa, fat hen, chickweed, and tulips [36,37]. The occurrence of *Turnip mosaic virus* (TuMV, *Potyvirus* genus, Potyviridae family) was confirmed in *Rorippa prostrata*. TuMV infects a wide range of cruciferous plants worldwide including economically important vegetable, oilseed, biofuel, forage and ornamental crops [38,39]. It is transmitted non-persistently by more than 40 different aphid species and has a wide natural host range including a wide range of weed species [40]. The occurrence of different viral communities in weeds growing nearby the agriculture landscape indicate the possibility of a spillover from the reservoirs to other hosts. This study confirmed that weeds can harbour viruses between seasons, raising questions about how to mitigate the risk that weeds contribute to disease outbreaks in crops. These questions are highly relevant, as some contemporary management strategies (i.e., organic farming, and practices aimed at increasing biodiversity in and around farmlands) potentially may also affect the intensity of viral spillover between crops and wild plants. Interestingly, the presence of Tomato black ring virus (TBRV) (*Nepovirus* genus, Secoviridae family) was also confirmed using HTS in *Sambucus nigra* plants growing nearby zucchini crops. The genomic RNAs of TBRV were associated with subviral satellite RNAs. The occurrence of TBRV in zucchini crops has been previously reported [41]; however, there is no evidence for a direct association between the infection of wild plants and zucchini crops. The origin of the virus within these zucchini crops remains unclear; there are no data regarding seed transmission, and the possibility of transmission by nematode vectors has not been verified yet.

HTS has had a major impact on the analysis of diversity and evolution of virus populations [42,43,44]. In this growing field, a lot of new data have been generated that shed light on genetic variability, evolutionary dynamics, and connections between virus evolution and the host evolution and ecology [45]. Susi et al. [2] used small RNA sequencing to identify viruses in *Plantago lancelolata* populations in Southwest Finland. Aside from the previously reported Plantago lancelolata latent virus (PLLV), the authors found sequences representative of four new virus species: Plantago latent caulimovirus, Plantago betapartititvirus, Plantago enamovirus, and Plantago closterovirus. Moreover, they discovered variations in virus prevalence across *P. lancelota* populations and found that symptomatic plants were more likely to be infected than asymptomatic ones (84 vs. 44% infected). A metagenomics survey performed in 115 wild species growing in wild or anthropic habitats in central Spain revealed an infection caused by geminiviruses concentrated on 25 species growing in habitats next to crops and within them [46]. Geminiviruses constitute a large family of plant-infecting DNA viruses which have dramatically impacted agricultural yields over the past 50 years [47]. Geminiviruses are a major threat to the food security of developing countries in the tropical and subtropical regions of the world [48,49]. New, potentially divergent geminivirus species were also detected from uncultivated plants by Bernardo et al. [8] and further characterised by Claverie et al. [50].

Bernardo et al. [8] used the geometagenomics approach to analyse the prevalence and diversity of virus communities in cultivated and uncultivated areas in two Mediterranean climate regions: the Rhone delta in France and the Western Cape in South Africa. Overall, 1725 plant samples were collected in crops and wild plants in areas with different levels of human management and viral genomes were sequenced using 454 pyrosequencing. Virus prevalence and diversity were evaluated only at the family level and, on the basis of pairwise sequence comparisons, similarities related to virus sequences were grouped into operational taxonomic units (OTUs) at the family level for deeper analysis. Surprisingly, this study found that both virus prevalence and diversity were higher in agricultural areas. In this study, 94 previously unknown virus species were reported. The majority of these unknown viruses originated from uncultivated plants, supporting the notion that the presently known crop-infecting viruses represent just a relatively small fraction of all plant-infecting virus species. Interestingly, six OTUs from this study potentially represent highly divergent geminiviruses. Further studies conducted by Claverie et al. (2018) [50] identified six completely novel geminiviruses from four different South African uncultivated endemic plant species (*Euphorbia caput-medusae, Limeum africanum, Exomis microphylla,* and *Polygala garcinia*) and two from France (*Juncus maritimus* and cultivated species *Medicago satvia*)*,* thus revealing an unexpected degree of geminivirus diversity.

A high level of genetic variability was also shown in the study of Ma et al. [6] who simultaneously explored diversity of virus populations in a cultivated crop plant, tomato (*Solanum lycopersicum),* and a botanically related weed, *Solanum nigrum* (European black nightshade). The samples were collected from tomato and black nightshade growing side by side and HTS was used to determine composition and richness of the virome. A total of 20 viral families were discovered. Virome richness was highly variable, with tomato and nightshade sharing only 17.9% of OTUs. Overall, the results provide evidence for limited viral spillover between tomato and wild nightshade populations, with some exceptions. For example, *Broad bean wilt virus 1* (BBWV1, *Fabavirus* genus, Secoviridae family) was detected in six of the samples from black nightshade populations (out of 11 populations sampled), but it was not detected in any of the seven sampledtomato populations. Moreover, BBWV1 showed a large and unexpected level of genetic diversity, suggesting frequent reassortment between RNA1 and RNA2. As BBMWV1 has a relatively wide host range—including many crops such as pea, spinach, lettuce, pepper, and tomato—and is aphid-transmitted, its absence in these tomato populations suggests the existence of biological or epidemiological barriers that limited its spillover potential [6]. These studies highlight the importance of characterising the viromes of both crops and wild plants to understand the drivers shaping viral emergence and disease dynamics [8,51,52].

All of the above-mentioned studies explore the spatial variation between viral communities in weeds and wild plants; however, information on the temporal variation in wild plant virus communities is still very limited. Thapa et al. [31] analysed the effects of host identity, location and sampling year on the taxonomic composition of plant viruses in six wild plant species [*Ambrosia psilostachya* (*Asteraceae*), *Vernonia baldwinii* (*Asteraceae*), *Asclepias viridis* (*Asclepiadaceae), Ruellia humilis* (*Acanthaceae*), *Panicum virgatum* (*Poaceae*) and *Sorghastrum nutans* (*Poaceae*)] comprising 400 specimens of the target hosts collected from twenty sites in the Tallgrass Prairie Preserve in northeastern Oklahoma over four years. Samples were tested for the presence of plant viruses applying VANA and double-stranded RNA enrichment methods, allowing for comparisons of community composition based on the host species, location, or time of collection. This large-scale metagenomic study classified viral sequences into OTUs for a better assessment of virus diversity. Results indicated that host species had a significant effect on virome composition compared to the location and sampling time. The majority of plant viruses identified during the study were novel viral species that had not previously been reported.

HTS techniques can also be used to study different host–pathogen interactions [33]. By combining metagenomic and culturomic approaches, Ma et al. [33] assessed the richness, diversity, and composition of leaf-associated fungal and viral communities from pools of herbaceous wild plants. Samples were collected in four sites in southwest France corresponding to cultivated or natural ecosystems. Overall, 161 fungal families and 18 viral families were identified. The community composition of both fungal and viral populations showed strong site specificity. Ecosystem management had a significant effect on the microbial communities, as indicated by higher fungal community richness in natural ecosystems and a higher viral family richness in cultivated ecosystems, suggesting that leaf-associated fungal and viral communities are under the influence of different ecological drivers.

We have provided an overview of different studies that use HTS methods on wild plants and weeds in Table 1. When we examine the results of these different studies, are there any trends that become apparent? Firstly, all of these studies discovered novel viruses, highlighting the power of HTS methods for virus discovery and demonstrating how little virus biodiversity has been catalogued to date. Secondly, plant viruses were regularly found in healthy plants, often at high prevalence. These observations confirm that many plant virus infections are asymptomatic in weeds and wild plants, but also underline the importance of understanding both the economic and ecological implications of this type of infection. Moreover, studies on viral prevalence should take the asymptomatic nature of many infections into account and sample plants accordingly. Thirdly, there has been little evaluation and comparison of the different methods for meta-genomics described so far. We think there is a need to systematically compare methods and identify their limitations, as this will impact the interpretation of results. For the studies we considered, only Thapa et al. [31] compared methods extensively, and found marked differences between the viromes obtained with dsRNA to VANA-based methods. In contrast, other studies do report confirmation of HTS identification with other methods [2,34]. Due to publication bias, studies with negative results may be underrepresented, so these confirmations should be interpreted conservatively. Fourth, three studies [8,30,33] found that virus diversity was higher in agricultural than wild ecosystems. The two studies that estimated prevalence [8,30] also found a higher prevalence of plant virus infections in agricultural systems. On one hand, agricultural systems with low diversity and plants selected for crop yield, at the expense of other traits such as resistance to pathogens, might be expected to amplify viruses. On the other hand, one might expect wild ecosystems to harbour more viral diversity due to the higher host diversity present, if many infections are asymptomatic and have a low prevalence due to higher host diversity. As more metagenomics data become available, and as we learn more about the biases and sensitivity of different nucleic extraction and sequencing approaches, it will be extremely interesting to see whether this trend is confirmed and the extent to which it is found across different ecosystems. Finally, we note that the vast majority of studies have sampled only one or two times, and hence there is a pressing need for longitudinal studies. Here it is interesting to note that PCR-based detection of viruses over multiple years in wild *Arabidopsis halleri* populations shows seasonal variation in accumulation, whilst the prevalence and genome of the virus populations are surprisingly consistent [53].

Are there any general features of plant-associated viromes that can be tentatively identified, based on the studies reviewed here? First, there is the observation that diverse dsRNA viruses, a very large proportion of which appear to be novel, usually dominate the plant-associated viromes [8,31]. A second observation is that the richness in DNA viruses tends to be lower than that of RNA viruses, observing the balance between these two groups in the currently recognised taxonomy approved by International Committee on Taxonomy of Viruses [8].

### 2.3. Effect of Host Population, Climate Changes, and Land Use on Virus Emergence and Biodiversity

In natural ecosystems, host populations typically have greater genetic diversity and more complex age structures than populations in agroecosystems, particularly in regions where crops are grown as monocultures with low genetic diversity. In wild plant communities, by contrast, plant genomes are under natural selection. This may favour the persistence of multiple resistance alleles or result in selection for novel resistance alleles. Understanding the role of plant genetic diversity in controlling patterns of virus emergence is a long-standing goal of biological research, and it is central for the management of current infectious diseases and developing long-term strategies to prevent the emergence of new ones [54]. Increased use of nature areas for agriculture has resulted in significant losses in biodiversity and ecosystem simplification. Virus emergence in crops is a complex process that involves interactions among wild and crop hosts, insects, and other virus vectors, and changes in ecosystems. Although the data are quite limited, it is thought that reduced biodiversity in agroecosystems in terms of both plant species richness and the genetic diversity within species will lead to increases in the incidence of viral disease [5,54]. The loss of biodiversity would increase host abundance and density, which, in turn, would lead to higher transmission rates and a higher virus prevalence [55,56]. These conditions are likely to result in the evolution of higher virulence, as increasing the density of susceptible hosts is an important driver of the evolution of increased virulence [57]. Understanding whether there is a general relationship between biodiversity and disease risk is critical for projecting and reducing the impacts of future disease outbreaks. The relationship between biodiversity and disease risk has been analysed in Cereal yellow dwarf virus (CYDV) and Barley yellow dwarf virus (BYDV). CYDV and BYDV belong to the *Luteovirus* genus (Tombusviridae family) and infect a wide range of grass species [7]. CYDV and BYDV are generalists; however, they show partial host specialisation, as they accumulate at different levels in different grass species. Experimental studies on B/CYDVs in grasslands of the western US reveal that the prevalence and community compositions of B/CYDV is affected by interactions of host plants, vectors, vertebrate herbivores, as well as biotic and abiotic factors [7]. The interactions between B/CYDV, their aphid vectors, and host plants explain the effect of species composition on infection risk and underline the complexities of virus ecology and epidemiology [7,12,58,59]. These results also suggest that ecosystem simplification and biodiversity losses favour higher virus prevalence.

The opposite scenario has been shown in the study of Susi and Laine [30], who investigated the impact of land use on five virus species detected in 27 *P. lancelota* populations across the Aland Islands in Finland. They investigated whether the structure of virus communities differed between natural and agriculture edge settings. The study revealed that virus species richness declined with increasing plant diversity in a natural ecosystem, while in cultivated areas virus species richness was moderately higher, and not associated with plant richness. This difference was not explained by changes in host richness between these two habitats, suggesting potential virus spillover and increased transmission of viruses across the agroecological interface [30]. The effect of host richness on infection prevalence was negative and not altered by agricultural land use.

Some viruses capable of devastating crops and harvested products have become more active and damaging because their geographical ranges have expanded due to climate change. A changing climate can contribute to the successful spread of newly introduced viruses or their vectors, and the establishment of these organisms in new habitats that were previously unfavourable. Non-native plants and insects may also expand their geographic ranges and hereby invade communities where they were previously not present. These species might constitute reservoir hosts or vectors of plant viruses capable of causing epidemics in nearby crops [60]. In terrestrial ecosystems, studies have increasingly found evidence of virus infection in a broad range of wild plants [2,30]. Global climate change is one of the main reasons for the increased economic impact of aphids in temperate regions, which can adapt to new environmental conditions rather quickly [61,62]. The global temperature over the past century has been increasing by approximately 0.8 °C and is expected to rise between 0.9 and 3.5 °C by 2100. Such changes will not only have a great impact on the growth and cultivation of different crops but will also affect the reproduction, spread, and severity of many plant viruses [63]. Since pests and pathogens are currently responsible for more than 25% of crop and post-harvest losses, any range expansions or altered host–pathogen interactions that exacerbate damage and loss will have very serious implications for the security of the world’s food supplies [64]. Aphids generate both winged and wingless adult morphs, whereas whiteflies are all winged. Winged individuals are the ones that contribute the most to virus spread because of their ability to fly over long distances. The short developmental times and great capacity of reproduction of aphids make them especially suited to adapt to climate change [62,65]. Climate change can have various effects on vectors, such as the modification of a vector’s phenology, over-wintering, density, migration, and interactions with its natural enemies. Migration potential and long-distance dispersal of virus vectors may also be affected by changes in weather patterns. Increased winter temperature induces earlier starts to the aphids’ annual cycle, increasing the proportion of winged adults and stimulating their flight activity [66]. Furthermore, plant viruses are able to influence the behaviour and fitness of their vectors in such a way that changes in plant–virus–vector interactions can affect their transmission, although it remains to be seen that such changes are adaptive under natural conditions. It has been shown that aphid-transmitted Cucurbit aphid-borne yellows virus (CABYV; *Polerovirus* genus, Luteoviridae family) can induce changes in the alighting, settling, and probing behaviour of its vector, the cotton aphid *Aphis gossypii* [67]. CABYV infects cucumber, melon, squash, and watermelon and has also been detected in many weed species, which may be efficient reservoirs [68].

## 3. Problems to Be Solved and Future Directions

The efficacy of viral metagenomics to detect variable virus communities in weeds and wild plants relies on sampling, nucleic acid extraction, high-throughput sequencing, and bioinformatic analysis strategies. Each of these steps will pose its own challenges and may affect estimates of virus community composition.

### 3.1. Challenges in Virus Enrichment and Nucleic Acid Extraction

The choice between protocols using viral particle enrichment or total nucleic acid extraction protocol will affect the relative abundance of viral reads, as reviewed by Maclot et al. [69]. It is important to be aware of the bias that the chosen method will introduce prior to metagenomic sequencing. For example, we expect differences in the sensitivity of VANA and dsRNA-based approaches. VANA protocols enrich virus particles, and will thus not recover non-encapsidated viruses, viruses with low titers, or viruses with an unstable particle morphology. dsRNA-based approaches can also detect single-stranded RNA (ssRNA) and DNA viruses. dsRNA is a replication intermediate for ssRNA viruses, whereas for DNA viruses overlapping transcripts from ORFs in opposite orientations can hybridise to generate dsRNA [70,71]. However, for some ssRNA viruses, low amounts of dsRNA can be produced [72] and for DNA viruses successful detection will depend on the organisation of viral transcripts, hence this approach may not be suited for detecting many viruses that do not have dsRNA genomes. Ma [73] compared these two viral sequence enrichment approaches: dsRNA and VANA for plant virome analysis in complex pools representative of the most prevalent plant species in unmanaged and cultivated ecosystems. Additionally, a novel bioinformatic approach was implemented and virus richness was assessed by determining OTUs following the clustering of conserved viral domains [8]. To analyse virome diversity, six different sites were selected in southwest France and for each site, a total of 200 individual plant samples were collected in spring 2016. Analysis using either dsRNA or VANA metagenomic approaches revealed a high viral diversity in all sites, including the presence of novel dsRNA viruses. Generally, both approaches recovered largely the same viral families, however, DNA viruses were not efficiently recovered using dsRNA strategy. On the other hand, for the VANA approach, a low efficiency of detection was observed for virus families with low titer and/or less stable particles. Therefore, for researchers mainly interested in RNA viruses, a dsRNA-based approach was recommended as it provided a more comprehensive description of analysed (phyto)viromes [51,73].

Secondly, another critical step in plant virus metagenomics is the preparation of high-quality nucleic acid extracts. Polysaccharides, high levels of RNases, polyphenols, and fibrous tissues are often encountered in plant samples, and fibrous tissues such as lignin (wood) are difficult to break up and remove, which makes extracting high-quality nucleic acids challenging for wild plant species. Numerous adaptations have been made to nucleic acid isolation methods—including modifications to tissue storage, maceration, preparation and extraction—to obtain sufficient, high-purity nucleic acids for non-model plant species, depending on the tissue type and the presence of secondary metabolites and inhibitors [74]. These experimental details can be of vital importance, but are often not reported in sufficient detail for plant virus metagenomics work to be fully reproducible. We hope these omissions become less common due to fewer constraints on publication format and the possibility of including a wide range of supplementary materials, aided by many contemporary initiatives to increase scientific reproducibility and transparency. Given the broad pallet of nucleic acid approaches and techniques available, as well as the challenge extractions of many wild species pose, selecting the most appropriate approach will remain a hurdle that must be carefully managed.

### 3.2. Challenges in HTS and Bioinformatics

Analysing virus metagenomics data in weeds and wild plants is challenging, both conceptually and practically. Currently, the HTS method commonly used for viral metagenomics is Illumina sequencing. Illumina HiSeq and NextSeq instruments generate short, paired end reads of 2 × 75−150 bp in length, read from the short fragments produced during the library preparation process. Illumina MiSeq offers a considerably lower volume of reads, but read length can be doubled up to 2 × 300 bp paired end reads. From these short sequencing reads, longer virus genome sequences can be assembled using a variety of pipelines and software packages. After the assembly process, viral genomes can, in some cases, be incomplete (e.g., partial genomes or genome segments that are missing), or assembly can result in chimeric sequences (where reads from different genetic origins within a sample are similar and therefore erroneously assembled into one sequence). Chimeric assemblies are more likely more likely to happen when metagenomics are performed on pooled samples, which is common in plant virus metagenomics studies. In the future work, the problem of chimeras may be solved by using HTS methods which generate longer reads, such as Oxford Nanopore sequencing, or PacBio SMRT, or HiFi sequencing. Due to the small genome size of plant viruses, ranging from 4 to 30 kb [75], long read sequencing can capture entire genome segments in a single read. For plant viruses, long read sequencing has been used on geminiviruses [76], *tombusviridae* [77], and several potyviruses including Wheat streak mosaic virus [78], Plum pox virus [79] and Yam mild mosaic virus [80]. However, the host plants in these studies were all crop plants, with the exception of the geminiviruses found in *Medicago arborea*, a shrub in the *Fabaceae* family [76]. In these studies, a variety of DNA and RNA HTS library preparation protocols are used, and the choice of library preparation affects the virus detection sensitivity. Generally, viruses were reliably detected with long-read sequencing.

Although long read sequencing provides opportunities for plant virus metagenomics, there are a few drawbacks. Firstly, all library preparation protocols for RNA sequencing require poly-A tailed RNA as input, as sequencing adapters are designed to bind to this region. However, many plant virus species do not have a poly-A tail. This limitation can be circumvented by adding a poly-A tailing step prior to sequencing, but this reaction is not specific to viral RNA and will also affect any host RNA present in the sample [81]. Secondly, many of the RNA-based library preparation protocols do not allow for barcoding, lowering the throughput of samples that can be sequenced. For DNA viruses these constraints are not applicable. One key limitation for large-scale application of long read Nanopore sequencing is the requirement for higher quantity and purity of the nucleic acid input compared to other sequencing technologies such as Illumina, which is more difficult to achieve for wild plant species. Given that most of the long-read plant virus metagenomics have been performed in crops, it remains to be seen whether Nanopore sequencing is readily amenable to larger metagenomic surveys of plant viruses in weeds and wild plants.

While other microbial communities, such as bacteria or fungi, may be routinely characterised through a conserved gene (16S and ITS, respectively), viruses have no such conserved genomic regions due to their varied evolutionary history. Since there is no universal marker for viruses, viral genome segments are recognised through sequence similarity to other viruses. This results in several problems in identifying viral metagenomes from weeds. Firstly, virus genome databases are incomplete, and there is currently a bias towards plant viruses found in crops. This means that viruses that occur exclusively in wild plants may not be recognised due to a lack of sequence similarity with known viruses. The significant proportion of viral metagenomics data that has no detectable sequence similarity with any known biological entity has been referred to as “dark matter”, and its viral nature remains an open question [17,51]. The observations of many researchers indicate that even enriching specifically for viral nucleic acids associated with viruses by VANA or dsRNA purification leads to the detection of many viral sequences that cannot be readily assigned to species, either due to their novelty or the short length of the sequence [8,17]. This pervasive ambiguity indicates the need to create new and innovative algorithms for discovering viruses based not only on sequence similarity, but also to use conserved, informative genome regions or domains for the clustering of viral sequences or the functional protein motifs utilised by viruses for productive infection [82,83]. Nevertheless, the taxonomic assignation of the viral contigs identified from HTS data faces many challenges that have been reviewed in detail [82]. Given the large number of novel agents uncovered in metagenomics studies, the most widely used approach, BLAST-based annotation, generally provides unreliable results at the species and genus level and still has weaknesses at the family level [17,82]. These problems raise questions of whether and how metagenomic sequence data should be incorporated into the ICTV taxonomy. To potentially improve this situation, Simmonds et al. [82] proposed solutions for virus classification including creation of new virus species, assigning new species and genera to existing families, and improvement of the procedure for the classification of viruses. These problems are not unique to viruses, and perhaps inspiration can be derived from initiatives taken with other microorganisms. For example, many bacterial and archaeal species detected by metagenomics cannot be cultured. There is a convention in place on how to taxonomically classify these candidate species and further improvements on how to recognise the value of these genome sequences have been suggested [84,85].

Lastly, the analysis of virus metagenomics data is a practical challenge for non-specialised teams. Working with HTS-data requires computing equipment capable of storing and processing large datasets, as well as expertise on genome assembly analysis pipelines and packages. However, the decrease in its basic operational costs, further development of simple, reliable, and user-friendly bioinformatics tools for data analysis and sequence mapping will make the utilisation of metagenomics affordable to a larger group of researchers and phytosanitary services. Although virus detection using HTS is a powerful tool, in case of limited coverage across a viral genome and low sequencing depth, confirmatory tests may be required.

## 4. Concluding Remarks

In summary, metagenomics approaches have a huge potential for detecting viruses, characterising viromes and exploring virus emergence and epidemiology. Plant virus metagenomic studies have shown that viruses are common in weeds and wild plants, even in the absence of symptoms, and these studies have uncovered many new, uncharacterised viruses. These results highlight how powerful HTS approaches are for virus discovery, whilst also showing the spectacular amount of viral biodiversity harboured by wild plants. Nonetheless, HTS methods have also some pitfalls and several challenges remain in this field of study: choices regarding protocols for enrichment and sequencing of plant viromes will affect the virus species and viral genetic variation that can be uncovered. This can be achieved by the wider implementation of plant virus metagenomics studies, which have the capacity to detect viruses alone or in mixed infection and the potential to reveal the presence of novel or unsuspected viruses [17,86]. Key questions about the association between plant diversity and virus prevalence, as well as how the biodiversity–disease relationship changes in plant populations adjacent to agriculture areas, remain open. However, by combining HTS approaches with an ecological perspective on plant viruses, we can take important steps towards addressing these issues in earnest.

## Figures and Tables

**Table 1:** Studies using metagenomic approaches for virus detection and diversity in weeds and wild plants.

Sample Collection Year	Country	Host Plant	HTS Platform	HTS Template	Main Conclusions	Reference
2005–2008	USA	*Ambrosia psilostachya* *Vernonia baldwinii* *Asclepias viridis* *Ruellia humilis* *Panicum virgatum* *Sorghastrum nutans*	454	VANA and dsRNA	•Many unknown viruses identified in asymptomatic plants. •More viruses detected by dsRNA than by VANA. •Plant virus composition is highly variable. Of the factors investigated, host plant explains most but only a small fraction of this variation.	[31]
2010–2012	South Africa, France	Cultivated and un-cultivated plants along the interface of natural and agricultural ecosystems.	454	VANA	•94 unknown viruses were identified, primarily from uncultivated plants. •Highest prevalence and identified diversity of plant viruses found in agricultural ecosystems.	[8]
2012–2013	South Africa, New Zealand	*Arctopus echinatus* *Lolium perenne*, *Panicum ecklonii*, *Stipagrostis sp*	454, Illumina	VANA, and enrichment of circular DNA	•5 novel, circular DNA viruses were identified.	[32]
2013	Finland	*Plantago lanceolata*	Illumina	Small RNA of 20–30 nucleotides, mainly composed of small interfering RNAs.	•Virus prevalence and diversity varied among the sampled host populations. •Potentially novel virus species belonging to Caulimovirus, Betapartitivirus, Enamovirus, and Closterovirus genera were identified. •In a PCR-based follow-up study, viral prevalence and diversity were lower in natural ecosystems, suggesting spillover from agricultural to natural ecosystems.	[2]
2017	France	200 plants (representing 80 species) collected for each of 4 sites, with 2 sites being managed and 2 being unmanaged sites.	Illumina	dsRNA on both leaves and leaf- associated, cultured fungi	•161 fungal families and 18 viral families were identified. •A large viral diversity dominated by novel dsRNA viruses was detected. •At the family level, more diversity in the managed plots, as some ssRNA viruses are only present there. •Leaf associated fungal communities were more diverse in unmanaged systems.	[33]
2017–2018	France	*Solanum nigrum*	Illumina	dsRNA	•A total of 20 viral families were discovered. •The novel ilarvirus, here named Solanum nigrum ilarvirus 1 (SnIV1), was detected in both tomato and nightshade samples. •Virome richness was highly variable. Tomato and nightshade shared only 17.9% OTUs, indicating different viromes for close relatives at the same location.	[6]
2018–2019	Poland	*Verbena officinalis* L., *Silene latifolia Poir.* *Rorippa × prostrata* *Robinia pseudoaccacia L*	Illumina	Total RNA	•Detection of two viruses that had not been previously found in Poland: Clover yellow mosaic virus (ClYMV) and melandrium yellow fleck virus (MYFV).	[34]

## Data Availability

Not applicable.
